# Kounis Syndrome: A Case Report and a Review of Recent Literature

**DOI:** 10.7759/cureus.64627

**Published:** 2024-07-16

**Authors:** Maysan Almegbel, Khalid Alshamardl, Latefah Aleshaiwi, Fawaz Almutairi

**Affiliations:** 1 Internal Medicine, King Abdulaziz Medical City, Riyadh, SAU; 2 Adult Cardiology, King Abdulaziz Medical City, Riyadh, SAU; 3 Interventional Cardiology, King Abdulaziz Medical City, Riyadh, SAU

**Keywords:** st-elevation myocardial infarction (stemi), mi kounis, allergy and anaphylaxis, myocardial infarction, allergic mi, kounis syndrome

## Abstract

Kounis syndrome (KS) is commonly defined as acute myocardial infarction (AMI) secondary to exposure to an allergen. There are multiple identified allergens that are associated with KS, examples include medications, food, and contrast media. After exposure to an allergen, the allergic pathway is triggered leading to vasospasm in coronary vessels which later on presents as AMI. A high index of clinical suspicion is of crucial importance as there are multiple variants of KS. Each type requires a different management approach depending on the severity of the presenting symptoms. Here, we present a case of a 65-year-old female with a history of transient ischemic attack (TIA) who presented to our hospital with symptoms of urinary tract infection and received the first dose of ceftriaxone while in the ER. She then developed symptoms of shortness of breath, chest pain, and diaphoresis associated with overall skin itchiness with ECG evidence of ST-elevation myocardial infarction (STEMI) in the inferior leads. She was given initial measures to treat possible allergic reactions including steroids and diphenhydramine and her ECG showed complete resolution after that; therefore, she was presumed to have KS after exposure to antibiotics. In this case report, we elaborate more about our case and further explore management options for KS.

## Introduction

Allergic angina or acute myocardial infarction (AMI) secondary to allergen exposure is referred to as Kounis syndrome (KS) or allergic myocardial infarction, which was first described by Kounis and Zavras [1] in 1991. Multiple allergens were identified as a cause of KS including antibiotics, analgesics, and contrast media triggering a hypersensitivity reaction cascade and eventually causing vasospasm or plaque rupture [1-9]. This entity of vasospasm was observed in coronary arteries and was also reported to involve cerebral or mesenteric arteries [10,11]. Here, we report this case as possible KS secondary to ceftriaxone.

## Case presentation

A 65-year-old female with a past medical history of a prior transient ischemic attack (TIA), non-obstructing kidney stone, and bilateral knee osteoarthritis, with a 45-year history of smoking presented to the emergency department with a complaint of a one-week history of dysuria that was associated with subjective fever and shivering. At presentation, the patient denied any other symptoms of headache, dizziness, chest pain, or dyspnea and denied any history of allergies when asked. She was vitally stable, alert, conscious, and oriented with unremarkable physical examination. Upon arrival, her urine urinalysis was suggestive of urinary tract infection and her basic blood work was normal aside from leukocytosis. She then underwent a non-enhanced abdomen CT scan which showed a stable previously known non-obstructing ureteral stone with no other renal pathology.

**Figure 1:**
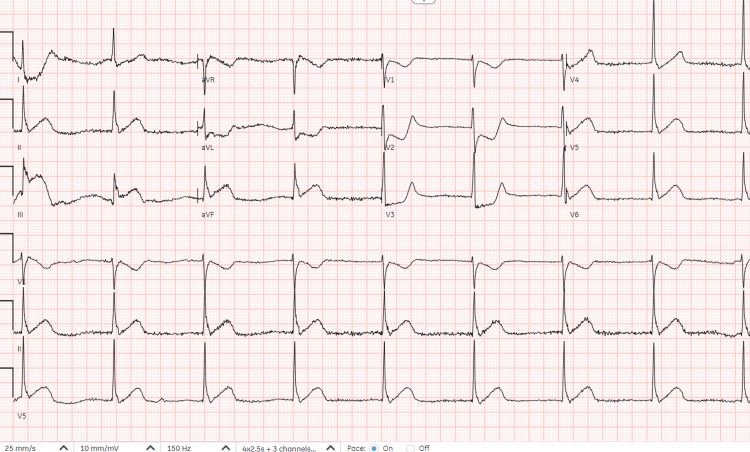
Initial ECG showing ST elevation in inferior leads with reciprocal changes.

**Figure 2:**
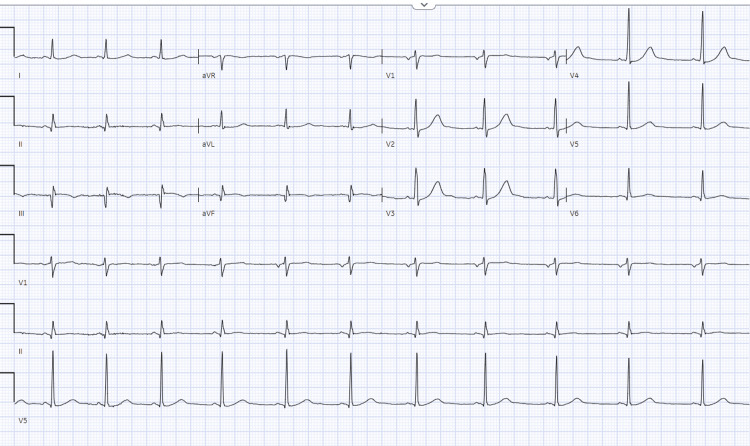
ECG after receiving diphenhydramine and hydrocortisone showed resolution of previous findings.

The patient was admitted as a case of a complicated urinary tract infection for intravenous antibiotics. Upon admission, the patient received IV ceftriaxone as an empiric therapy and after receiving it for approximately 30 minutes, she started to complain of chest pain, shortness of breath, and skin itchiness that quickly evolved into bradycardia with two episodes of vomiting. Her heart rate ranged from 57 to 32 beats per minute and her blood pressure was 126/78 with a respiratory rate of 26 breaths per minute. An electrocardiogram was done at the time of the episode and showed an ST-segment elevation in the inferior leads II, III, and aVF with reciprocal changes in leads V1-V2 and AVR (Figure 1). She was then shifted to a monitored bed and started on heparin infusion and dual antiplatelet therapy. She also received diphenhydramine and hydrocortisone upon developing these symptoms. A few minutes later, the patient started to stabilize vitally and her symptoms were improving. ECG was repeated and her ST elevation was completely resolved (Figure 2). Her cardiac enzymes were also obtained and came back negative.

She was kept under cardiac monitors with serial ECG and troponin levels. A further detailed history of allergies has revealed that she had a distant history of dyspnea, itchiness, and rash after taking ceftriaxone in another hospital. The patient was flagged as allergic to ceftriaxone and planned for elective cardiac catheterization the next day; however, the patient refused and was sent home against medical advice. We report this case as a possible KS due to ceftriaxone.

## Discussion

The development of acute coronary syndrome (ACS) secondary to exposure to an allergen is a rare condition. The association between allergic reactions and the development of ACS was first described by Kounis and Zavras in 1991 and referred to as KS [1]. The pathophysiology is still not fully understood; however, several hypotheses were proposed that several inflammatory mediators and cytokines such as histamine, tryptase, platelet-activating factor, leukotrienes, and thromboxane, that are secreted by mast cells following hypersensitivity reaction playing a major role causing coronary vasospasm or contributing in the rupture of already existed atheromatous plaque [3,4].

There are three variants of KS have been described. Type I variant being observed in a patient with no cardiovascular risk factors and normal coronary arteries in which the symptoms are caused by coronary artery spasm (CAS) due to acute release of inflammatory mediators which lead to either a transient CAS with no elevated cardiac enzymes or CAS that lead to AMI. The type II variant developed in patients who already have coronary artery disease in which plaque ruptures as a result of allergic reactions that lead eventually to AMI. Lastly, the type III variant is defined as AMIs that happen due to drug-eluting stent thrombosis in which the thrombus removed showed several materials secreted by mast cells [4,5]. A review study by Abdeghany et al. [2] summarized 175 published cases of KS and reported type 1 as the most common type followed by types 2 and 3 respectively. The limitation in our case is lacking of cardiac catheterization. However, mast cell-mediated transient coronary spasm due to ceftriaxone is the most possible cause (type 1 variant) for several reasons. First, the onset of allergic and cardiac symptoms started 30 minutes after administration of ceftriaxone. Secondly, the spontaneous resolution of the ST segments elevation and the patient's cardiac symptoms without percutaneous coronary intervention (PCI). Thirdly, normal cardiac enzyme results.

Several allergens have been identified as triggers causing KS including food such as shellfish, some pharmaceuticals such as analgesics, proton pump inhibitors, contrast media, and antibiotics especially penicillins as they are the most common antibiotic observed to cause KS [2]. Moreover, there are three reported cases of ceftriaxone as a causative agent one of which was reported in Saudi Arabia [6]. Ceftriaxone is a third-generation cephalosporin that is widely used in treating several infections, and it has been known to cause several hypersensitivity reactions with various clinical presentations of urticaria, angioedema, or even anaphylactic shock [7,8]. With that being said, KS might be more common than we think and probably is underdiagnosed due to the inability to recognize it and mislabeling a patient as simply anaphylaxis or allergy case.

When diagnosing a patient with KS, a high index of clinical suspicion with good knowledge is required to guide investigations and management. A thorough detailed allergic history is key in preventing such a presentation. The clinical picture can range from slowly progressive symptoms of skin manifestation with itchiness and urticaria to symptoms of ACS with chest pain, perspiration, shortness of breath, and sometimes syncope [4,9]. A thorough history of previous allergic reactions and their clinical manifestations should be obtained. Ordering basic investigations such as chest X-ray, ECG, and cardiac enzymes should be initiated as soon as the patient develops these symptoms along with the administration of steroids and anti-histamines to manage the acute phase of allergy with co-administration of anticoagulants and anti-platelets to manage angina and possible ACS [9,2]. Cardiac enzymes are most likely to be negative in type 1 KS but ECG findings such as ST-segment elevation corresponding to the involved coronary artery and reciprocal changes, any degree of heart block, T wave changes, or arrhythmias including bradycardia or atrial fibrillation can be found [4,9]. Moreover, as reported by Abdeghany et al. [2], the most common ECG finding in KS was ST-segment elevation, especially in the inferior leads. Likewise, our patient developed bradycardia with ST-segment changes in the inferior leads corresponding to the area supplied by the right coronary artery.

Further investigation towards diagnosis includes coronary artery catheterization and more recently cardiac magnetic resonance imaging (CMRI) which can be used to confirm the diagnosis of KS [4,9]. Coronary catheterization is of importance as it can distinguish between the presence of CAS, atherosclerotic changes, or the presence of a thrombus causing myocardial infarction. It can also show the presence of other cardiac wall abnormalities such as ballooning of the heart apex found in Takusubo cardiomyopathy which can co-exist with KS [4,9]. Moreover, CMRI has been proven valuable in type 1 KS as dynamic CMRI will show normal washout in the delayed contrast-enhanced images for the sub-endocardial area involved in KS [9,12]. Another investigation that has been used more recently is single photon emission computed tomography (SPECT) using thallium-201 and 125I-15-(p-iodophenyl)-3-(R,S) methylpentadecanoic acid (BMIPP) SPECT in cases of type 1 KS. The SPECT result can demonstrate myocardial ischemia [4]. Both CMRI and SPECT can be positive despite having normal coronaries during coronary catheter angiography [4,9].

As a rare disease, the evidence behind proper management guidelines is lacking. Therefore, management of KS depends on the type of KS and largely on managing both allergy/anaphylaxis symptoms and possible ACS simultaneously. It is however challenging to proceed with anaphylaxis/allergy management in patients who may be already on cardio-selective medications for ACS or heart failure such as beta-blockers. These medications may interfere with the optimal effect of epinephrine when given for anaphylaxis [4,13]. On the other hand, corticosteroids used as a part of allergy management may have an unfavorable outcome in ACS patients as they may affect myocardial wall healing [9,13]. A balanced approach is therefore of crucial importance.

In type 1 KS (vasospastic with pro-inflammatory mediators release), administration of antihistamines and corticosteroids can control mild-moderate allergic reactions and intramuscular (IM) adrenaline in more severe allergy/anaphylactic symptoms [4]. Management approach in type 1 KS follows the guidelines for anaphylaxis measures with fluid resuscitation as needed as well [9]. Angina symptoms can be managed with sublingual nitroglycerin and calcium channel blockers; however, both medications should be given with caution as they may worsen hypotension associated with anaphylaxis and may cause angioedema themselves [4,9]. In type 2 KS (allergic MI), management initially is similar to that of type 1 KS in addition to MI management. ACS protocol guidelines should be followed which include beta-blockers and morphine. The risk of using beta-blockers in KS is that it can worsen the vasospastic effect of adrenaline and thus the use of glucagon may be necessary [4,9,13]. In type 3 KS (allergic stent thrombosis/restenosis), management includes all the previously mentioned steps of allergy management and ACS protocol in addition to the removal of intra-stent thrombosis if possible with the placement of a new stent [4,9]. In summary, the management of KS focuses on targeting elements involved in allergy and myocardial ischemia. Our patient improved dramatically with normalization of her ECG findings after administration of only IV diphenhydramine and hydrocortisone.

## Conclusions

KS, defined as allergic MI, is an uncommon medical condition encountered in medical practice. Possibly due to the complexity of the disease process that involves both allergy and myocardial ischemia, guidelines in KS management are still lacking. A balanced approach focusing on both entities of the disease while keeping in mind the underlying pathophysiology of the three possible types of KS is currently the standard of care. A high clinical suspicion is of great importance in managing such a delicate and possibly life-threatening disorder.
